# Effectiveness of virtual reality-based toothbrushing instruction on oral health outcomes: A randomized controlled trial

**DOI:** 10.1007/s00784-026-06819-6

**Published:** 2026-03-17

**Authors:** Hye-Jin Kwon, Seung-Hee Ryu, Eun-Jae Choi, Jung-Ah Lee, Seon-Jip Kim, Hyun-Jae Cho

**Affiliations:** 1 Department of Preventive Dentistry & Public Oral Health, School of Dentistry and Dental Research Institute, Seoul National University, 101 Daehak-ro, Jongno-gu, Seoul, 03080 South Korea; 2 Sue & Bill Gross School of Nursing, University of California, Irvine, CA USA; 3 Department of Dental Hygiene, College of Health Science, Dankook University, 119 Dandae-ro, Dongnam-gu, Cheonan, 31116 South Korea

**Keywords:** virtual reality, randomized controlled trial, oral health education, plaque index, toothbrushing

## Abstract

**Background:**

Oral diseases, significantly driven by dental plaque, remain a major global health concern. Traditional toothbrushing instruction (TBI) methods are often limited in interactivity and long-term skill retention. Recent digital innovations, particularly virtual reality (VR) offer highly interactive educational experiences. This randomized controlled trial (RCT), therefore, aimed to evaluate the effectiveness of VR-based TBI compared to traditional instruction using plaque-disclosing agents.

**Methods:**

This single-blind, two-arm RCT was conducted at a school of dentistry. Healthy adults aged 20–39 years were recruited and randomly allocated using a computer-generated randomization sequence to either a VR group or control group. The VR group received a structured toothbrushing curriculum delivered through immersive VR (Meta Quest 3), whereas the control group received one-on-one instruction from a dental hygienist using plaque-disclosing agents. The primary outcome was the change in plaque index (PI) from baseline to the two weeks follow-up, and secondary outcomes was oral health knowledge-attitude-behavior (KAB) scores. Participants were blinded to group assignment; however, the investigators who performed the assessments were not.

**Results:**

30 participants were randomized (15 in each group), and all participants were included in the final analysis. The VR group demonstrated a significantly greater mean reduction in PI compared with the control group (0.44 ± 0.48 vs. 0.07 ± 0.26; *p* = 0.012). Both groups showed improvements in KAB scores. Notably, the VR group achieved significantly greater gain in oral health attitude (39.06 ± 4.80 vs. 38.30 ± 3.66; *p* = 0.008) and behavior (4.73 ± 1.39 vs. 4.53 ± 1.30; *p* = 0.004). No harms or unintended events such as dizziness or discomfort was reported.

**Conclusion:**

Immersive VR toothbrushing education improved plaque control and enhanced oral health attitudes and behaviors compared with traditional instruction. This approach may also address the limitations of conventional education, including reliance on professionals and restrictions of time and place, and holds promise for broader public health applications. Further research should examine long-term effects and scalability.

**Trial Registration:**

: KCT0010914 (Clinical Research Information Service [CRIS]); retrospectively registered on August 19, 2025.

## Introduction

Oral diseases remain a significant global public health issue, affecting approximately 3.5 billion people worldwide and imposing considerable burdens on healthcare systems [1]. Conditions such as dental caries, severe periodontal disease, and tooth loss substantially impair quality of life across all age groups [2, 3]. A key etiological factor underlying these diseases is dental plaque, a microbial biofilm adherent to tooth surfaces. If undisturbed, plaque shifts toward pathogenic bacterial dominance, directly causing gingival inflammation and periodontal destruction [4–7]. Effective management of dental plaque through consistent and precise toothbrushing is essential for preventing oral diseases and maintaining optimal oral health [8–12].

However, achieving adequate plaque control remains challenging, partly due to limitations inherent in traditional oral health education methods. They often relies on verbal instructions, static visual aids, and pamphlets [13–15]. While these methods have long been foundational to oral hygiene education, growing evidence highlights their notable limitations [16], including passive delivery, insufficient engagement, and poor retention of skills, resulting in limited long-term effectiveness [17, 18].

Recent advances in digital technology, particularly VR, offer promising alternatives to overcome these limitations [19]. VR provides interactive and immersive learning environments, enhancing engagement and facilitating effective skill acquisition through realistic simulations [20, 21]. It enables repeated practice of essential oral hygiene behaviors in a controlled setting [22]. Dentistry has explored VR extensively for reducing dental anxiety and pain [23–28], enhancing professional training [29–32], and promoting oral health education [33–35].

A recent systematic review identified 5 VR-based oral health education studies [36], highlighting several limitations. Most VR interventions target children or adolescents [23, 35, 37], with adult studies mainly involving those with disabilities [38] or healthcare assistants for geriatric populations [39]. Studies targeting healthy adults remain scarce, limiting their broad applicability to general oral hygiene education. Furthermore, most VR studies primarily assess subjective outcomes via questionnaires or interviews [23, 38, 39], which may inadequately capture actual clinical changes. Finally, despite many studies employing RCT designs, control groups are often absent [23], receive no intervention [38, 39], or involve weak comparisons like verbal instructions [34], potentially inflating perceived effectiveness. Additionally, existing VR education predominantly employs passive viewing methods through VR headsets.

To address these limitations, this study aims to evaluate the effectiveness of VR-based TBI on oral hygiene improvement in adults. A RCT design is employed with a robust control group receiving traditional instruction using plaque-disclosing agents. Clinical indices (Plaque index and bleeding on probing) and behavioral measures (knowledge-attitudes-behaviors survey) comprehensively assessed. Our VR instruction was developed to leverage unique educational affordances, facilitating active user engagement, realistic practice, and immediate feedback to enhance toothbrushing skills and promote behavioral change.

## Methods

This study was designed and reported in accordance with the CONSORT 2010 guidelines [40].

### VR-based toothbrushing instruction content

VR-based TBI content was developed through iterative consultation and testing by a team consisting of dentists, dental hygienists, and a content development company (X2R, Asan, Republic of Korea). The software was developed using the Unity engine (Unity Technologies, San Francisco, USA). Hardware implementation was based on Meta Quest 3 platform (Meta, Menlo Park, California) with controllers serving as virtual hands to grasp toothbrushes, select menu options, answer quizzes, and perform toothbrushing movements. The VR environment resembled a powder room, one wall presented a 3D dentition model with synchronized instructional animation and narration; the opposite wall featured a virtual mirror, enabling users to directly practice learned brushing techniques on virtual teeth reflected in the mirror. Users could easily alternate between these two areas by simply turning their heads or repositioning themselves within the virtual space.

Curriculum consisted of 4 sequential modules:

#### Brushing technique

**Fig. 1:**
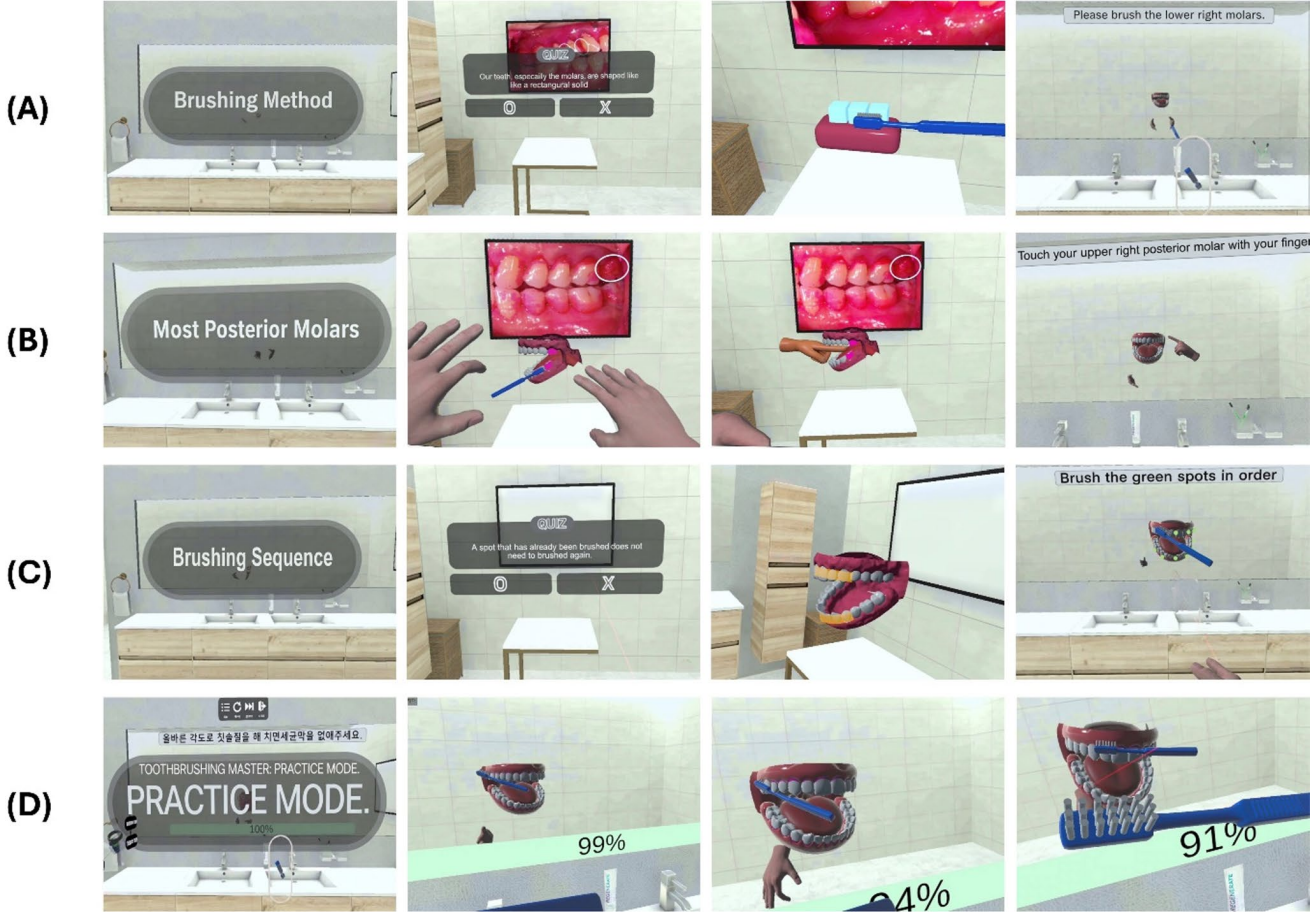
Interactive toothbrushing education modules covering technique instruction and simulation practice

Introduction to Bass method [41], instructing user to tilt the toothbrush at a 45-degree angle toward gingival margin for effective plaque removal (Fig. 1 (A)).

#### Most posterior molars

Guidance on thorough brushing of most distal molars, which are often overlooked (Fig. 1 (B)).

#### Brushing sequence

Users were guided to follow a systematic brushing sequence to ensure complete oral coverage and avoid redundancy or omission (Fig. 1 (C)).

#### Toothbrushing Master session

Final module provided a self directed brushing session using a virtual mirror. Pink linear plaque was distributed along gingival margins of all teeth and were removed only when user accurately executed Bass technique with a 45-degree angle. Simulation started with a 100% plaque gauge that decreased as correct brushing was performed, allowing participants to practice previously learned brushing techniques (Fig. 1 (D)).

### Participants and randomization

A priori power analysis was performed using G*Power 3.1.9.4 (Heinrich-Heine-Universität Düsseldorf, Germany) [42]. Assuming a medium effect size = 0.25, 80% power, and α = 0.05, a total sample size of 28 was needed. Participants were recruited through an online research announcement, and those who volunteered underwent screening based on predefined eligibility criteria.

#### Inclusion criteria

Adults aged 20–39 years; individuals with ≥ 24 natural teeth (excluding third molars); natural teeth were counted by excluding teeth lost, replaced with implants, or restored with crowns, veneers, laminates, or extensive onlays involving smooth surfaces; and those capable of independently operating a toothbrush.

#### Exclusion criteria

Individuals with uncontrolled systemic diseases were excluded. Uncontrolled systemic diseases were defined as medical conditions that were not medically stable or adequately managed at the time of screening. These conditions included uncontrolled disorders of the cardiovascular, metabolic, neurological, or other systemic categories that were considered to pose a potential safety risk or to interfere with participation in VR-based oral health tasks.

Additional exclusion criteria included acute dental needs or planned scaling within 2 weeks, vestibular or motion-related symptoms, limitations in manual dexterity, regular use of electric toothbrushes, and current use of orthodontic appliances. Individuals who normally wear glasses and could not substitute them with contact lenses due to incompatibility with the VR headset were also excluded.

Participants were randomly assigned (1:1) to either the VR group or the control group using Microsoft Excel (Microsoft Corp., Redmond, WA, USA).

### Experimental protocol

**Fig. 2:**
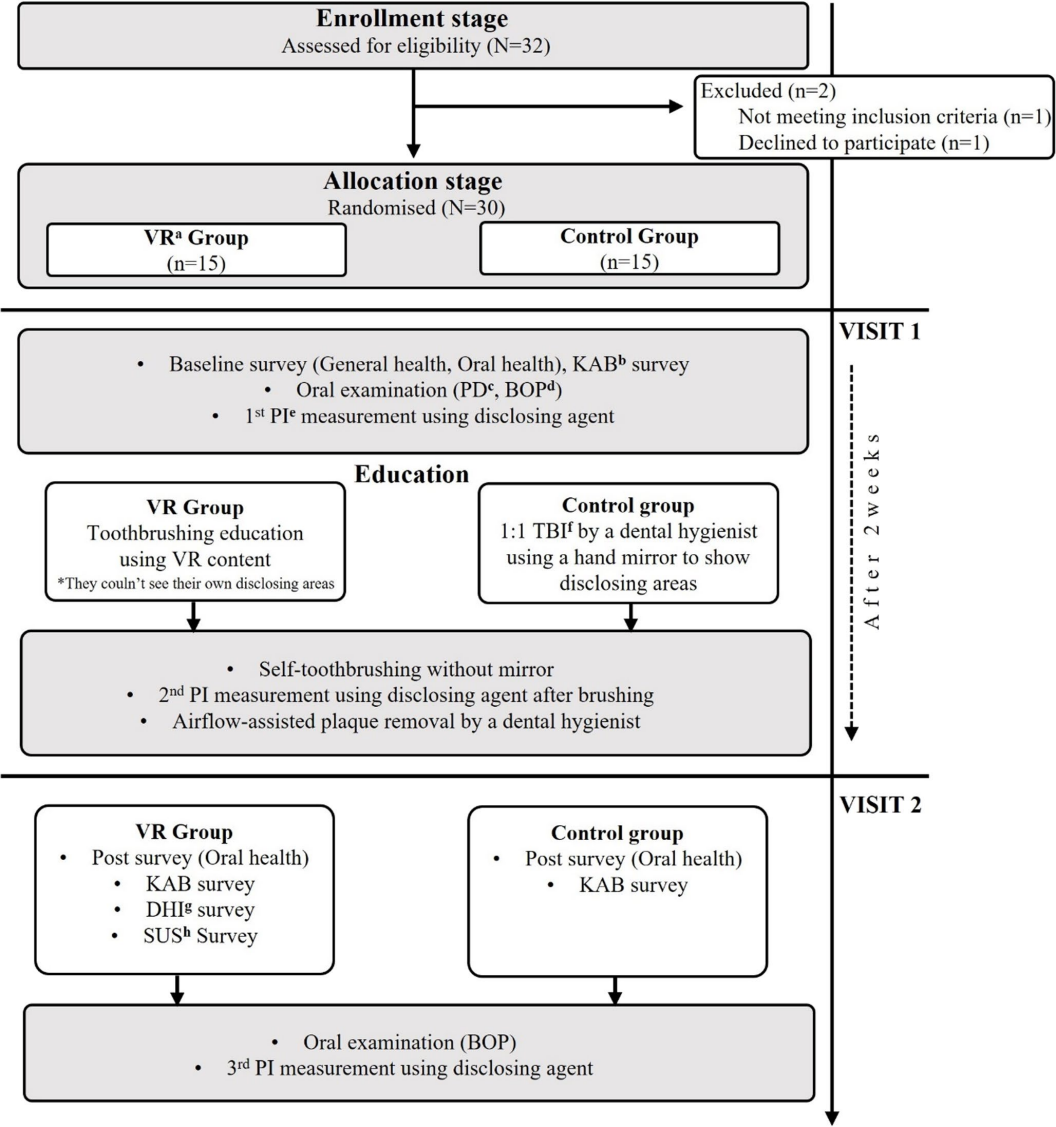
Experimental protocol and participant flowchart, ^a^VR: virtual reality, ^b^KAB: knowledge-attitude-behavior, ^c^PD: periodontal depth, ^d^BOP: bleeding on probing, ^e^PI: plaque index, ^f^TBI: toothbrushing instruction, ^g^DHI: dizziness handicap inventor, ^h^SUS: system usability scale

A single-blind, two-arm RCT was conducted at Seoul National University School of Dentistry (Seoul, Republic of Korea) over a one-month period in January 2025. Figure 2 illustrates the experimental protocol integrated with the participant flowchart.

#### Pre-visit instructions

All participants were instructed to refrain from toothbrushing for 6 h and from food intake for 2 h before each visit [43]. They were also asked to avoid using oral hygiene aids from 2 days before the first visit.

#### First visit

At the first visit, all participants completed surveys assessing sociodemographic characteristics, oral health status, and oral health knowledge-attitudes-behaviors. Clinical oral examinations were conducted, measuring periodontal pocket depth, bleeding on probing (BOP) and PI using a plaque-disclosing agent (Trace, Young Innovations, USA).

**Fig. 3:**
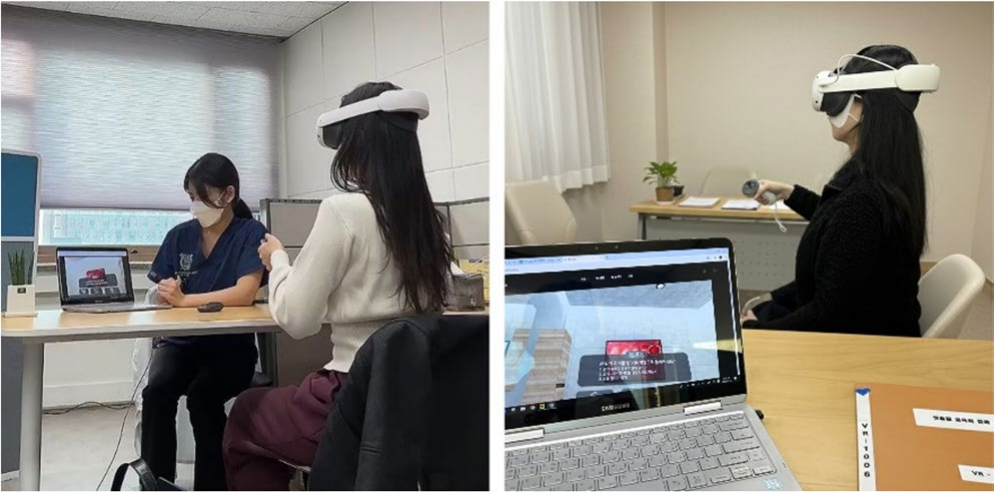
Toothbrushing education using virtual reality in the virtual reality group

Participants in the VR group received toothbrushing education using VR content, with minimal intervention from the researcher unless assistance was required with equipment operation or technical issues (Fig. 3). The VR intervention consisted of a sequential curriculum designed to provide the same intended educational content to all participants. The hands-on component required completion of a predefined mission, and the actual duration varied slightly depending on individual familiarity with the VR equipment. Overall, the average education time was approximately 5 min. They then performed toothbrushing without a mirror. They were strictly restricted from viewing their own disclosed plaque throughout the entire procedure, including during toothbrushing, in order to avoid any bias. Immediately after education, a second PI measurement was performed.

Participants in the control group received one-on-one toothbrushing instruction from a dental hygienist. Instruction was individualized based on each participant’s plaque, but the focus on heavily covered areas and common errors, as well as the duration, were consistent at approximately 5 min. They also brushed their teeth without a mirror, followed by the second PI measurement.

After completing these procedures, all participants underwent professional mechanical tooth cleaning using an Airflow device (EMS, Nyon, Switzerland) to standardize oral hygiene status before the follow-up period. All participants used a standardized soft bristled manual toothbrush (3-row, end-rounded nylon) and a 996-ppm fluoride toothpaste provided by the investigators.

### Intervention

During the two weeks, participants were instructed to brush their teeth as taught during the educational session. They used their usual toothpaste and toothbrush. Use of any oral hygiene aids and all dental visits, including scaling, were prohibited.

#### Second visit

Two weeks later, during the second visit, all participants completed follow-up surveys regarding oral health status and oral health KAB. The VR group additionally completed surveys evaluating satisfaction with the VR content and dizziness symptoms. Clinical examinations were repeated for all participants, assessing BOP and conducting a third PI measurement.

### Clinical outcomes

#### Plaque index

PI was evaluated using Turesky modification of the Quigley-Hein Index [44], scoring plaque accumulation from 0 to 5. Each tooth was divided into mesial, middle, and distal sites, assessed separately on buccal and palatal (or lingual) surfaces, for a total of 6 sites per tooth. Score were recorded at 6 sites per tooth, excluding teeth that were missing or otherwise not evaluable.

#### Bleeding on probing

BOP was assessed using a UNC periodontal probe at the same 6 sites per tooth with presence of bleeding recorded as 1, and absence as 0, across all available sites of the 28 teeth.

### Questionnaires

Demographic characteristics, general health behaviors were collected (Visit 1 only). Oral health behaviors survey assessed self-perceived oral health condition, toothbrushing frequency, brushing sequence, oral hygiene aids usage, and history of dental visits (Visits 1 and 2). KAB survey was adapted from previous studies [45] and translated into korean to enhance participants’ comprehension. It included 8 knowledge items, 9 attitude items and 7 behavior items. VR group additionally completed dizziness handicap inventory (DHI) [46] survey and a modified 20-item system usability scale (SUS) [47] survey.

### Blinding

Participants were blinded to their group assignment. Recruitment materials and instructions employed neutral language, describing the study as a “Comparison of various TBI methods” to avoid revealing allocation details. Specifically, participants in the VR group were informed that they would experience one of several educational methods, including VR, without explicit mention of their experimental status. VR equipment was concealed from the control group throughout the study procedures. However, blinding of investigators was not feasible due to limited research personnel.

### Data analysis

All statistical analyses were performed using SPSS Statistics version 19.0. (IBM, Armonk, NY, USA) Continuous variables were expressed as mean and standard deviation, and independent T-tests were performed to evaluate between-group differences. Categorical variables were presented as percentages and analyzed using Chi-square tests. To assess both time-dependent changes and interaction effects between groups, repeated measures ANOVA was employed for PI, BOP and KAB scores. Where appropriate, Bonferroni corrections were applied for post-hoc analyses to control for type I error. Differences between groups in changes of PI from baseline to the 2 weeks follow-up were assessed using independent T-tests. For the KAB survey, knowledge and behavior items, which had Yes or No responses, were scored as 1 point for correct answers and 0 points for incorrect answers. Attitude items were assessed using a 5-point Likert scale ranging from 1 to 5. Negatively worded items were reverse-scored before summation. Reliability analyses were conducted for the KAB and SUS survey using Cronbach’s alpha. P-value of less than 0.05 was considered indicative of statistical significance throughout all analyses.

### Ethical considerations

Ethics approval was granted by institutional review board of seoul national university school of dentistry (IRB No. S-D20230019). Participants were informed that they could withdraw from the study at any time without any negative consequences, and that any potential side effects associated with participation were fully explained in advance.

## Results

### Demographic and oral health characteristics

A total of 32 participants were recruited; 1 did not meet inclusion criteria and 1 declined participation. The remaining 30 participants were enrolled and randomized equally into 2 groups (*n* = 15 each).

The demographic and oral health characteristics of the patients are shown in Table 1. The mean age was 24.93 (SD 3.49) years, and no significant age difference was found between groups (VR: 23.93, SD 2.19; Control: 25.93, SD 4.28; *p* = 0.12). No significant group differences were found in personal income, education level, exercise habits, alcohol consumption, or smoking status. Regarding daily toothbrushing frequency, participants reported brushing once per day (1/30, 3%), twice per day (21/30, 70%), or three times per day (8/30, 27%), with similar distributions across groups (*p* = 0.46). Interdental brush (3/30, 10%) and floss usage (12/30, 40%) were also distributed between groups (*p* = 0.68 and *p* > 0.99).

**Table 1:** Demographic and oral health characteristics of participants

Characteristics	Control group(n=15)	VR^a^ group(n=15)	Total(N=30)	p-value
Age (year), mean (SD)	25.93 (4.28)	23.93 (2.19)	24.93 (3.49)	0.12
Sex, n (%)				
Male	7 (47)	8 (53)	15 (50)	0.72
Female	8 (53)	7 (47)	15 (50)	
Personal income, n (%)				
None	4 (27)	5 (33)	9 (30)	0.56
Low	6 (40)	3 (20)	9 (30)	
Middle low	2 (13)	4 (27)	6 (20)	
Middle high	3 (20)	2 (13)	5 (17)	
High	0 (0)	1 (7)	1 (3)	
Education level, n (%)				
High school	5 (33)	6 (40)	11 (37)	0.71
≥University	10 (67)	9 (60)	19 (63)	
Exercise, n (%)				
Yes	7 (50)	5 (33)	12 (40)	0.39
No	7 (50)	10 (67)	17 (57)	
Alcohol consumption, n (%)				
Yes	9 (60)	11 (73)	20 (67)	0.44
No	6 (40)	4 (27)	10 (33)	
Smoking, n (%)				
Smoker	1 (7)	2 (13)	3 (10)	0.72
Non-smoker	12 (80)	12 (80)	24 (80)	
Ex-smoker	2 (13)	1 (7)	3 (10)	
Tooth brushing, n (%)				
Once/day	1 (7)	0 (0)	1 (3)	0.46
Twice/day	11 (73)	10 (67)	21 (70)	
Thrice/day	3 (20)	5 (33)	8 (27)	
Current use of interdental brushing, n (%)				
Yes	3 (20)	0 (0)	3 (10)	0.68
No	12 (80)	15 (10)	27 (90)	
Current use of flossing, n (%)				
Yes	6 (40)	6 (40)	12 (40)	0>.99
No	9 (60)	9 (60)	18 (60)	

^a^VR virtual reality

### Clinical outcomes

#### Plaque index

At baseline, PI were comparable between groups (Control: 2.94, SD 0.25; VR: 2.92, SD 0.34) (Table 2). Immediately post-intervention, both groups demonstrated a reduction in PI (Control: 2.34, SD 0.26; VR: 2.12, SD 0.35). At the 2 weeks follow-up, PI increased slightly in both groups but remained below baseline levels (Control: 2.87, SD 0.38; VR: 2.47, SD 0.58). A significant difference in PI change from baseline to the 2 weeks follow-up was found between groups (Control: 0.07, SD 0.26; VR: 0.44, SD 0.48; t_28 = 2.68, *p* = 0.012) (Table 3). Repeated measures ANOVA indicated significant changes in PI over time within groups (*p* = 0.024).

**Table 2:** Plaque index at baseline, after education, and 2 weeks follow-up by group, mean (SD)

Variables	Baseline	After education^a^	Follow-up at 2 weeks	*p*-value^b^
Plaque index				0.024
Control group	2.94 (0.25)	2.34 (0.26)	2.87 (0.38)	
VR^c^ group	2.92 (0.34)	2.12 (0.35)	2.47 (0.58)	

^a^Measured at the first visit, immediately after education,^b^Repeated measures ANOVA,^c^VR virtual reality

**Table 3:** Plaque index difference by group, mean (SD)

Variables	Plaque index difference^a^	T test (df)	*p*-value^b^
Control group	0.07 (0.26)	2.68 (28)	0.012
VR^c^ group	0.44 (0.48)		

^a^Difference: Baseline plaque index mean - After two weeks plaque index mean,^b^Indepentdant t- test,^c^*VR* virtual reality

#### Bleeding on probing

There was no significant change in BOP within or between groups after 2 weeks (Table 4). BOP remained unchanged in both groups (Control: from 0.04 to 0.04; VR: from 0.05 to 0.05; *p* = 0.785).

**Table 4:** Bleeding on probing at baseline and 2 weeks follow-up by group, mean (SD)

Variables	Baseline	Follow-up at 2 weeks	*p*-value^a^
Bleeding on probing			0.785
Control group	0.04 (0.04)	0.04 (0.04)	
VR^b^ group	0.05 (0.09)	0.05 (0.11)	

^a^Repeated measures ANOVA, ^b^VR virtual reality

### Questionnaires

Reliability analyses indicated acceptable internal consistency for the KAB (Cronbach α = 0.65) and modified SUS survey (Cronbach α = 0.91). No participants reported dizziness symptoms, including mild cases. After 2 weeks follow-up, both groups showed improvements in KAB scores (Table 5). Oral health knowledge score increased in both groups (Control: from 7.00 to 7.40; VR: from 6.90 to 7.53), but this change was not statistically significant (*p* = 0.583). Oral health attitude score significantly increased in the VR group (from 35.40 to 39.06) compared to the control group (from 37.70 to 38.30; *p* = 0.008). Oral health behavior score significantly increased in the VR group (from 2.53 to 4.73) compared to the control group (from 3.87 to 4.53; *p* = 0.004).

**Table 5:** Oral health knowledge, attitude, and behavior scores at baseline and 2 weeks follow-up, mean (SD)

Variable	Group	Baseline	Follow-up at 2 weeks	*p*-value^a^
Knowledge	Control group	7.00 (1.30)	7.40 (1.12)	0.583
	VR^b^ group	6.90 (1.16)	7.53 (0.74)	
Attitude	Control group	37.70 (3.08)	38.30 (3.66)	0.008
	VR group	35.40 (5.36)	39.06 (4.80)	
Behavior	Control group	3.87 (1.25)	4.53 (1.30)	0.004
	VR group	2.53 (1.25)	4.73 (1.39)	

^a^Repeated measures ANOVA, ^b^VR virtual reality

## Discussion

### Principal results

#### Clinical outcomes

At the 2-week follow-up, the VR group demonstrated a significant reduction in PI compared to the control group (*p* = 0.024). In the VR environment, participants repeatedly practiced toothbrushing using a virtual dentition model. This approach aligns with VR’s unique educational affordances, such as ‘heightened realism’ and ‘reflective observation’, enabling an integrated experiential learning cycle. A previous study on VR-based medical communication training similarly emphasized that systematic educational elements—particularly immediate feedback, clear protocols, and reflective practice—are critical of successful educational outcomes [48]. Thus, we speculate that our results stem from effectively leveraging VR’s unique affordances, rather than the mere use of VR technology.

No significant differences in BOP were observed. This might be explained by the relatively short intervention period, as gingival inflammation typically requires sustained plaque control over longer. Previous studies indicate that short-term interventions are less likely to show measurable gingival improvement [49].

#### Questionnaires

Oral health knowledge score increased slightly in both groups, but the change was not significant (*p* = 0.583). Participants in both groups had relatively high baseline (Control: 7.00; VR:6.90; maximum of 8 points), suggesting limited room for further improvement. Additionally, our intervention primarily targeted practical toothbrushing skills rather than comprehensive oral health information.

The improvement in attitude score in both groups may partly reflect the Hawthorne effect and social desirability bias. Despite the use of a powerful motivational methods—directly visualizing plaque on their own teeth—in the control group, the VR group showed greater improvement in attitude scores. This indicates that VR-based education can be as effective as real-world motivational techniques.

Behavior score improved significantly more in the VR group (*p* < 0.001). Traditional TBI methods involve either practice directly on one’s teeth, making accurate assessment difficult, or practice on dental models, which lack realism and self-directed engagement. In contrast, VR instruction combines realistic oral simulation and clear observational capability, enabling active and repeated practice, which likely promoted actual behavioral change.

### Comparison with Prior Work

Previous studies on VR-based interventions generally indicate positive impacts on oral hygiene outcomes, though findings vary depending on methodology and target populations.

According to a RCT involving 90 female high school students, participants who received VR-based oral health education showed significant reductions in both plaque (*p* < 0.01) and gingival indices (*p* = 0.017), compared to those receiving verbal instruction or no intervention [34]. This finding closely aligns with our results, suggesting VR’s potential effectiveness in improving clinical oral hygiene outcomes.

According to a study among children aged 9–12 years, VR oral hygiene education significantly increased flossing (*p* < 0.001), tongue brushing (*p* < 0.001), and daily toothbrushing frequency (*p* < 0.001) [23]. However, similar to our findings, oral hygiene knowledge score did not significantly improve (*p* = 0.550).

VR education was also examined by subdividing preschool children into immersive, semi-immersive, non-immersive VR, and traditional control groups. The immersive VR group showed significantly better behavioral outcomes compared to the other groups (*p* = 0.004), supporting our hypothesis that specifically leveraging immersive VR affordances enhances educational effectiveness [35].

A recent systematic review of 9 studies involving immersive technology education concluded that VR consistently improved oral health knowledge, attitudes, self-efficacy, and hygiene behaviors [36]. These conclusions are consistent with our findings, providing additional support for the effectiveness of VR instruction in improving oral hygiene behavior and plaque control.

Overall, previous literature supports VR-based oral health education as effective; however, outcomes vary by population and educational approach. Our findings add further evidence that carefully designed immersive VR interventions leveraging interactive, self-directed practice may offer clear advantages for improving oral hygiene compared to traditional methods.

### Strengths and Implications

This study has several strengths worth noting. First, our VR-based TBI content was carefully developed by a professional dental team, focusing specifically on improving practical toothbrushing skills through user-interactive content. This approach differs from previous interventions that often relied on passive observation of VR videos or limited interactions. Another practical advantage of VR education is that, unlike conventional methods requiring direct involvement from dental professionals, the VR method can be delivered independently once the hardware and software are set up. Thus, this approach has potential scalability for clinical or public health contexts. Furthermore, we selected a robust motivational control method (TBI with plaque-disclosing agents) commonly used in clinical settings, rather than conventional dentiform models or video-based education. Demonstrating superior effectiveness over this widely adopted clinical method highlights the practical value of our VR intervention. Methodologically, we utilized a RCT design with participant blinding, providing objectivity and reliability. To our knowledge, this is the first study to comprehensively evaluate both objective clinical outcomes (PI, BOP) and KAB scores following VR-based TBI in an adult population. This combined assessment offers deeper insights into how VR education influences not only clinical outcomes of oral health but also related knowledge, attitudes, and behaviors, providing valuable implications for future educational interventions.

### Limitations

There are several limitations to this study. First, the small sample size and short observation period limit the statistical power and generalizability. Second, although participants were blinded, examiners were not fully blinded due to practical constraints, introducing a risk of measurement bias. Third, the VR intervention requires specialized hardware and software, which can be cost-prohibitive, restricting accessibility and scalability. The high cost of equipment is recognized as a barrier to implementing VR in health education broadly [50, 51]. Additionally, there is a potential for VR-induced side effects, such as dizziness or nausea [26]; however, even the mildest levels of dizziness were not reported in our study.

## Conclusions

In this study, we compared VR-based TBI with traditional TBI using plaque-disclosing agents. The VR intervention significantly improved plaque control, oral health behaviors, and attitudes among adults. Although short duration limited measurable changes in gingival inflammation, these results suggest that the educational affordances of VR, including interactivity, immediate feedback, and self-directed practice, could effectively enhance oral hygiene outcomes. Future research should include larger participant groups, longer follow-up, and rigorous blinding to confirm these findings.

## Data Availability

The dataset generated and analyzed during the current study is not publicly available due to institutional and ethical considerations. Access to the data may nonetheless be granted on a case-by-case basis upon reasonable request and subject to the discretion of the corresponding author.

## Abbreviations

CONSORT: consolidated standards of reporting trials
VR: virtual reality
3D: three dimensional
IRB: institutional review board
PD: periodontal depth
BOP: bleeding on probing
GR: gingival recession
PI: plaque index
TBI: toothbrushing instruction
UNC: university of north carolina
KAB: knowledge-attitude-behavior
DHI: dizziness handicap inventor
SUS: system usability scale
